# The Coronavirus Pandemic: The Most Disruptive Force to Affect My Life and Practice

**DOI:** 10.1093/asjof/ojaa019

**Published:** 2020-05-09

**Authors:** Farzad R Nahai

Shut the office down? Words that prior to mid-March were unthinkable, then became murmurs and rumors, and then finally became a reality. Having been in practice going on 16 years, the coronavirus pandemic has been the most disruptive force that I have experienced on both my personal and work life. I lived through the 9/11 attacks as a general surgeon working on the Navajo Indian reservation and the financial crisis of 2007-2008 as a fairly new plastic surgeon just 3 years into private practice. While those events were historic with both short-term and long-term ramifications on my practice and my personal life, they both fall short of what the current pandemic is doing to all of us. We are living the short-term effects right now, what we do not know yet is what the long-term effects of the pandemic will be.

Shut down? Yes, shut down. That disruption became our reality on March 16th, almost a month prior to this being written. Disruption in our field and in society is generally attributed to new technology that displaces old tech (think Uber versus taxis, the electrification of automobiles) and is generally seen as change that is positive. The coronavirus pandemic has been disruptive to an extensive and deep degree with for the most part negative effects; however, there have been some positives that have come from this.

Before I get into the details of how the pandemic has affected my practice, here is a brief overview of my situation. I am a partner in a private practice multi-specialty group with 7 partner physicians, 5 associate physicians, 7 mid-level providers (PA’s and nurses), 4 locations in the Atlanta area, and a total of 122 employees as of March 1, 2020. Leading up to us closing our office and a statewide announcement for nonessential businesses to close and citizens to stay at home, we were meeting as partners almost daily in preparation for what we thought would be inevitable, shutting down the practice. So, once the order came, we were not caught totally off guard and had already discussed how things were going to go. It went something like this; cancel all elective visits and procedures immediately, see only essential patients (immediate post-ops, those with wounds), and perform only necessary cases (pending cancer reconstructions with open wounds and major reconstructive cases at the hospital). At the same time, my group of partners met daily for 2 hours on the phone discussing the details of our financial position and what we were going to do with our staff as we were (and still are) facing down closing the office indefinitely. Frequent meetings were also occurring with managers, staff, and our attorneys. One of the most assuring things we did early was an analysis of our weekly and monthly expenditures as it compared with our cash on hand, accounts receivable, and line of credit. As employees are the largest chunk of expenditure and we were looking at a closed office, we made an early effort at deciding who our “A” group of staff was, the group we considered indispensable.

All our efforts were and continue to be able to put ourselves in a position that we would all have a place to go back to after this passes. We were preparing for the worst possible situation; running out of money to keep the practice afloat long enough to weather a pandemic storm. That required a lot of hard decisions to be made in short order all geared toward saving as much money as possible for the practice. All accounts payable were held immediately, all partners agreed to not take pay for April, minimal skeleton crews were run to finish off the week and see the urgent and emergent patients, and planning soon began to incorporate telemedicine into our practice. With a large group, and the extra time on our hands, many of our efforts occurred simultaneously and we continued to meet on the phone daily to keep everyone up to date.

Leading up to the government’s announcement of the Cares Act and the Paycheck Protection Program (PPP) we continued to prepare for the worst which included letting go of a large number of our staff. As it became evident that the PPP was going to be a reality, a significant part of our efforts was focused on educating ourselves about it and preparing the application. We had our “B” list of employees ready but ended up not having to make the very hard decision to let them go because of the PPP. No matter your political leaning, the PPP saved many of our employees’ jobs and there is something to be said for that. Until our loan is in hand, we are relying on the cash we have with our line of credit as a backup and minimizing all expenses.

As the preparations were being made for our financial security, at the same time, we were preparing the office to run as safely and efficiently as possible. The telemedicine I mentioned earlier was quickly incorporated into the practice. That required training providers and staff and patients for the that matter on to use it properly, efficiently, and within the guidelines set forth by the American Medical Association and the insurance carriers. While not a major revenue generator and not applicable to all patients (the elderly are reluctant, and I think it remains to be seen how much an aesthetic practice can do via telemedicine), it at least keeps us engaged with our patients and small bit of cash flowing in. Even though we have been ostensibly closed and directing patients to telemedicine, in reality, we have running in “limp mode.” Each physician who has essential patients to see (urgent and emergent visits only, no elective visits) runs a skeleton crew 1 or 2 days a week following a 15-point safety protocol. Highlights of that protocol include: (1) streamlining appointments to decrease the number of patients in our facility at any given time, (2) calling all patients the day before their scheduled appointment and pre-screening for recent travel, cold symptoms, flu-like symptoms, fever, cough, and difficulty breathing, (3) providing gloves and masks to all patients upon entering the facility and further screening them for any ill symptoms, (4) keeping all doors open throughout the facility to minimize the need to touch any door handles or doorknobs and book patients one at time spaced apart so that no patients are in the waiting area, (5) implementing enhanced sanitation and disinfecting processes in exam rooms, in-between patients, and (6) requiring all medical staff to wear gloves, masks, and personal protective equipment (PPE) that will be changed after every patient encounter. Again, we have a 15-point protocol in place, I have just included some of the principle aspects.

To date, none of our providers or staff have become ill (save one scare that happened before we closed) and many of our patients have been very appreciative of our efforts. It has not been easy nor has it been without its stressful moments. Any staff with fever or cough have been told to stay at home for 2 weeks, no exceptions, unless cleared by a negative coronavirus test (which have been hard to come by). A handful of patients became very difficult to handle when they were informed of our policies resulting in them putting their selfish and myopic tendencies on display. The same went for a small minority of our staff members whose behavior wrote their own letter of release. This was one of the positives of the situation; the pandemic helped us weed out some of the staff that perhaps we needed to let go anyway. While we all feel for those losing their job at a time like this, strictly enforcing our policies, even to the point letting an employee go, does honor and confirm the good behaviors of the rest of the staff who value their job and value the practice.

Staring down an indefinite closure not only creates stress for the obvious reasons at work, but it also carries over to our personal lives. If you have kids (I have 2), they are at home remote learning and very likely not seeing any friends at all. Boredom sets in and previous restrictions on screen time and video games are loosened up to help cope with the situation, but this does lead to other behavior problems that have to be managed. On the plus side, my kids now do laundry, fill and empty the dishwasher, vacuum their rooms, and have a much better understanding of some of what it takes to run a household. For that matter, I do as well! I love my wife and kids dearly, but being home all day is difficult and I have had to find ways to manage. I have taken up project, after project, after project to help keep my sanity, and it does help but I am not made to stay at home indefinitely, so it is not ideal. One day, I had to go on a 4-hour drive on my own just to get out of the house. We keep up to date on the news, but the constant barrage of COVID-19 news can bring you down and make your stress worse, so you have to create breaks in consuming news. Having said all that, I have never had more time with my kids, I have never had more time to exercise, some of the home projects have brought me joy, and most of all I have been reminded how much I love my work and enjoy my practice and cannot wait to get back. This pandemic has created disruption like nothing we have seen before but with preparation, perseverance, and determination we can all pull through. Take care and stay safe.

